# Supplementary material for: Genomic characterization of clinical and environmental isolates of Bacillus anthracis in Peru
Source: PLoS Negl Trop Dis. 2026 Jan 23;20(1):e0013940. doi: 10.1371/journal.pntd.0013940 (PMC12854437; doi:10.1371/journal.pntd.0013940)
Supplement: Attachment — Submitted filename: Response to Reviewers.pdf [file pntd.0013940.s002.pdf]

Response to Reviewers

Title: Genomic Characterization of Clinical and Environmental Isolates of *Bacillus anthracis* in Peru.

Ref: PNTD-D-25-00853

Date: 10/01/2026.

To the Editors and Reviewers of PLOS Neglected Tropical Diseases

We would like to thank you for the opportunity to revise our manuscript. We have carefully reviewed the comments provided by the Editors and the Reviewers. We appreciate the constructive feedback, which has helped us to improve the quality and clarity of our work.

Below, we provide a point-by-point response to the comments. We have addressed the formatting requests and editorial requirements. Regarding the requests for additional wet-lab experiments (AST) and expanded phylogenetic analysis, we have acknowledged these as limitations in the revised Discussion, as current funding and time constraints prevent us from conducting further experimental work at this stage.

Changes in the manuscript are highlighted in the "Track Changes" version submitted.

JOURNAL REQUIREMENTS

1. Manuscript Source File: Response: We have uploaded the manuscript as a .docx file as requested.

2. Trademark Symbols: Response: We have removed all instances of trademark/copyright symbols (e.g., ®) throughout the text, specifically on pages 3 and 4 (e.g., QIAamp DNA Mini Kit, Qubit dsDNA, Intel Xeon).

3. Figure Files: Response: All main figures have been uploaded as separate files in .TIFF/.EPS format, following the journal guidelines.

4. Tables: Response: Tables are included in the manuscript file as editable, cell-based objects and have been removed from the list of separate uploads.

5. Copyright and Map (Figure 1): Response: We have confirmed that the map in Figure 1 complies with PLOS's CC BY 4.0 license. The map was constructed using open-source base layers. Specifically, reference departmental boundaries of Peru were obtained from the Peruvian National Open Data Platform (ODC-BY license), and country boundaries of South America were obtained from Natural Earth (public domain). We have updated the Figure 1 legend in the manuscript to include the direct links to these sources and their licensing terms.

6. Financial Disclosure: Response: We have updated the Financial Disclosure statement in both the submission form and the manuscript. We clarify that the funding provided by the Vicerrectorado de Investigación y Posgrado of the Universidad

Nacional Mayor de San Marcos (Grant B2410003m) supported the acquisition of the computational workstation, which was essential for performing the genomic analyses reported in this study.

RESPONSE TO REVIEWERS

Reviewer #1

Comment 1 (Introduction): The authors presented data from publications on the number of cases and outbreaks. This information could be improved with data from official government agencies with the number of current cases in humans and animals. What is the current number of anthrax cases in Peru?

Response: We appreciate the reviewer's crucial suggestion to include updated official data. We have performed a thorough search of government records and updated the Introduction accordingly. We have incorporated data from the Ministry of Health (MINSA) and the National Agrarian Health Service (SENASA), covering the period from 2000 to 2024 (412 cases) and highlighting the recent re-emergence of the disease. Specifically, we added information regarding the 2025 outbreak in Cerro Azul (Lima) with 6 confirmed human cases up to epidemiological week 40, associated with infected livestock, and the subsequent vaccination campaigns across coastal departments. has been added in lines 63–71. New references [09–11] have been added to support these findings.

Comment 2 (Methods): For the phylogenetic analysis, what was the criterion for choosing the 35 strains for genome comparison? Why were no strains from South America included? It would be interesting to compare them with the genomes of isolates from South America, in order to improve the phylogenetic analysis.

Response: We fully agree that comparing our isolates with genomes from South America would be highly relevant for improving the understanding of regional phylogeography. The 35 genomes included in the phylogenetic analysis were selected to represent the major *Bacillus anthracis* lineages (A, B, and C), encompassing a broad range of geographic origins, isolation sources, and time periods, as well as well-characterized reference strains commonly used in global phylogenetic studies. This selection strategy was intended to provide a robust global framework for contextualizing the Peruvian isolates.

However, the availability of complete *B. anthracis* genomes from South America in public repositories remains very limited. An exhaustive search of publicly accessible databases identified only a single representative genome of Brazilian origin, which was insufficient to support a robust regional phylogenetic reconstruction or to derive meaningful epidemiological inferences regarding pathogen circulation in South America. Therefore, the analysis focused on placing the Peruvian strains within a global

phylogenetic framework using well-established representative lineages, allowing for a sound and appropriate comparative interpretation consistent with the scope of the study.

Comment 3 (Results): Although described in the methodology, the SNP results were not demonstrated. These results could help in a more specific phylogenetic analysis of the clade.

Response: We thank the reviewer for this comment. In response, we have now added the SNP results to the Results section by incorporating a reference-based SNP analysis using Snippy v4.6 with the Ames Ancestor genome as reference (lines 233–235). A summary of core genome SNP counts and variant types for each isolate is provided in Supplementary Table S1, which supports a more detailed and specific phylogenetic interpretation of the A.Br.003/004 clade.

Comment 4: The delimitations of the analyses were not clearly described.

Response: We thank the reviewer for this comment. To address this point, we have added a dedicated Limitations section at the end of the Discussion, where we clearly describe the main constraints of the study.

Comment 5 (General): Suggestion for improving the complete characterization of the lineages that could assist epidemiological surveillance, since there is a different cluster and the detection of several resistance genes: Inclusion of Antimicrobial susceptibility testing for comparison with the results found in the analyses for AMR genes to verify their functionality. Expand the phylogeny analyses by comparing with genomes from South America: Argentina, Chile, Bolivia and Brazil (where a subclade was described). Include SNP analyses.

Response: We sincerely thank the reviewer for these valuable suggestions, which would undoubtedly strengthen the manuscript. While whole-genome sequencing allowed us to identify key antimicrobial resistance and virulence determinants, the performance of additional phenotypic antimicrobial susceptibility testing (AST) and the re-analysis of a large-scale regional phylogeny were not feasible within the current funding framework and study timeline. To address this point transparently, we have added a dedicated **Limitations** paragraph in the Discussion section (lines 323–330), where we explicitly acknowledge that our conclusions are based on genomic predictions and emphasize that phenotypic validation and broader regional phylogenetic comparisons represent important next steps for future research.

Reviewer #2

Comment 1: The objectives are clearly articulated and the study design is appropriate. The *B. anthracis* outbreak samples were clear describe from their origin and the year of

isolation. Being a whole genome sequencing study, there was no requirement to calculate a sample size and the relevant analysis was DNA extraction and amplification, Whole genome sequencing and Bioinformatics analysis. This was described well in the methods section.

Response: We thank the reviewer for the positive evaluation. No changes were required in response to this comment.

Comment 2 (Results): The results are clear and illustrative and are consistent with the described methods. The whole genome sequencing, phylogenetic analysis and the analysis of the plasmids was well described and illustrated. There is also clarity on the antimicrobial resistant genes identified. The tables, images and figures are clear and exhaustive.

Response: We thank the reviewer for the positive evaluation. No changes were required in response to this comment.

Comment 3: The discussion and conclusion is consistent with the finding on the genomic sequencing and this study adds to the body of knowledge and will assist in genomic surveillance during outbreaks and in general practice. The author could have been more overt on any limitations that may arise from the study. This study will guide in the prevention and management of Anthrax in Peru and contributes to the understanding of genotypic expression of *B. anthracis* in the South American region and globally.

Response: We thank the reviewer for this positive and insightful comment. In response to the suggestion, we have now explicitly addressed the main limitations of the study by adding a dedicated Limitations paragraph at the end of the Discussion, where constraints related to phenotypic validation and the limited availability of regional genomic data are clearly acknowledged (lines **323–330**).

Comment 4: The overall study is of sound quality as it is the first study to characterize the genome of *B.anthracis* in Peru from 2005 – 2017 from outbreak samples. The genome studies have shown high homogeneity of the samples, presence of antimicrobial resistance genes and also analysed plasmids PX01 and PX02 showing virulence potential of the bacterial. It is a very important study as it sheds light on the source and potential distribution of this bacterial that has been used for bio terrorism and biological weaponry.

Response: We thank the reviewer for this positive evaluation and for recognizing the relevance of our findings for genomic surveillance and public health. No changes were required in response to this comment.

Reviewer #3

Comment 1:

1) Yes, the objectives are clearly stated.

2) Yes, the study design is sufficiently appropriate.

3) Yes, the population is clearly described; it comprises a set of *B. anthracis* isolated from two major parts of Peru.

4) The authors have stated no specific hypothesis, and in my opinion none is needed for this study. The study is a survey of *B. anthracis* collected in Peru using the whole genome sequencing approach, and as such, I think it can stand on its own without a hypothesis.

5) Yes, the extent of statistical analysis needed is appropriate.

6) There seem to be no ethical or regulatory concerns; in any case, if there are any Peruvian, WHO, or US regulations applicable to this study, then I am not aware. In my opinion there are none, as long as the bacterial strains are properly safeguarded.

Response: We thank the reviewer for this comprehensive and positive evaluation of the study. No changes were required in response to this comment.

Comment 2 (Results): Yes, to all three questions. However, citing of Table 1 and the four figures within the narrative texts of Results and Discussion are scant, as they appear only once each. Figure 4 is not cited anywhere in the textual narrative. I suggest the authors cite the table and figures more frequently; once is not enough.

Response: We thank the reviewer for this helpful comment. In response, we have revised the Results and Discussion sections to increase the frequency of citations to Table 1 and all figures, thereby strengthening the connection between the textual narrative and the presented data. Table 1 is now explicitly cited in the Results and Discussion (lines 248 and 319), and Figure 4 is cited in the text at lines 198 and 306. These revisions were made to improve clarity and guide the reader more effectively through the results.

Comment 3 (conclusions): Yes, to all four questions.

Response: We thank the reviewer for this positive and comprehensive evaluation. No changes were required in response to this comment.

Comment 4:

1) Italicize bacterial names wherever they occur.

2) Cite the table and four figures more often; it would help the readers understand and pin the textual narratives with the data presented in the form of figures.

3) Because the authors have the complete genomic sequences of all *B. anthracis* samples, they should mention likely functionality of the ABR and virulence genes they mention. I think it should be feasible to determine from the gene sequences whether they are functional. If the sequences match the known wild type sequences, including those of the noncoding regions, then I think the authors can reasonable say that the genes and their products are likely functional. They may even add a table that lists names of the key genes in one column and any sequence deviations they found in the next column. They could add some relevant comments as footnotes regarding each sequence deviation and its functional implications. Doing so in my opinion would make their manuscript stronger

Response: We thank the reviewer for these helpful suggestions. Bacterial species names have now been consistently italicized throughout the manuscript. In addition, the Results and Discussion sections were revised to strengthen citations to Table 1 and Figures 1–5; in particular, Figure 4 and Table 1 are now explicitly cited in the text. We also expanded the Discussion to comment on the likely functionality of the identified antimicrobial resistance and virulence genes, based on their high similarity to well-characterized reference sequences, while noting that phenotypic validation is required in some cases. We believe these revisions strengthen the manuscript

Comment 5: The authors report a whole genome survey of *B. anthracis* samples collected in two regions of Peru. Their method was whole genome sequencing. The study is important, and it adds to the vast repertoire of *B. anthracis* sequencing data. The manuscript is nicely written, and it neither exaggerates, nor diminishes the significance of the findings. My major suggestion, which I mentioned above is that they should address whether, based on the sequences, any or all of these genes have the sequences that result in their products. If they found sequence deviations, then they should appropriately comment on those.

Response: We thank the reviewer for this helpful comment. We have expanded the Discussion to address the likely functionality of the identified antimicrobial resistance and virulence genes based on sequence similarity to well-characterized reference genes. Where relevant, we also note that gene presence does not necessarily imply phenotypic expression and that experimental validation would be required. These points are now clearly addressed in the revised manuscript.

We hope that the revised manuscript is now suitable for publication in *PLOS Neglected Tropical Diseases*.

Sincerely,

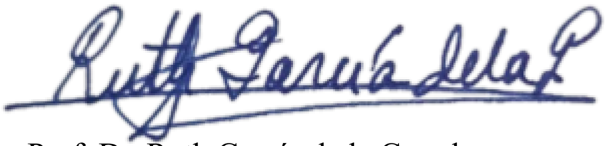

Prof. Dr. Ruth García de la Guarda
Laboratory of Molecular Microbiology and Biotechnology
Faculty of Biological Sciences, Universidad Nacional Mayor de San Marcos, Lima 15081, Peru.
